# HCV infection-associated hepatocellular carcinoma in humanized mice

**DOI:** 10.1186/s13027-015-0018-9

**Published:** 2015-07-27

**Authors:** Zhao Wang, Ningbin Wu, Abeba Tesfaye, Stephen Feinstone, Ajit Kumar

**Affiliations:** Department of Biochemistry and Molecular Medicine, The George Washington University, Washington, DC 20037 USA; Division of Viral Products, Center for Biologics Evaluation and Research, FDA, Bethesda, MD 20892 USA; Biochemistry and Molecular Medicine, The George Washington University, Ross Hall room 232, 2300 Eye Street, N.W., Washington, DC 20037 USA

**Keywords:** HCV Infection-associated HCC

## Abstract

**Background and Aims:**

Hepatitis C virus (HCV) infection is a major risk factor for chronic hepatitis, cirrhosis and hepatocellular carcinoma (HCC). Our aim is to explore molecular changes that underlie HCV infection-associated HCC in a humanized mouse model, in order to identify markers of HCC progression.

**Methods:**

Liver proteins from human hepatocyte-engrafted and HCV-infected MUP-uPA/SCID/Bg mice were compared with either uninfected controls or HCV-infected but HCC-negative mice by Western blotting. MicroRNA markers of HCC positive or uninfected mouse liver were analyzed by RT-PCR.

**Results:**

We describe the depletion of tumor suppressor proteins and induction of oncoproteins and oncogenic microRNAs (oncomiRs) in HCV-infection associated HCC. Similar depletion of PTEN protein in both HCC-positive and HCV-infected but HCC-negative liver suggests that PTEN depletion is an early, precancerous marker of HCC. By contrast, induction of oncoprotein cMyc, oncomiRs (miR21, miR221 and miR141) and inflammatory response proteins correspond to HCC progression.

**Conclusions:**

*While the l*oss of PTEN is important for the initiation of HCV infection-associated HCC, PTEN depletion by itself is insufficient for tumor progression. Liver tumor progression requires induction of oncoproteins and oncomiRs. Overall, human hepatocyte-engrafted (MUP-uPA/SCID/Bg) mice provide a suitable small animal model for studying the effects of oncogenic changes that promote HCV infection associated HCC.

## Introduction

Hepatocellular carcinoma (HCC) is the fifth most common cancer worldwide; its incidence is increasing because of the prevalence of chronic hepatitis C and B viral infections [1, 2]. The mechanism of HCV infection-associated HCC, however, is not well understood. To gain an understanding of the oncogenic changes that underlie HCC progression, we contrasted the expression levels of oncogenic proteins and microRNAs in liver tissues of a humanized mouse model of HCV infection-associated HCC [3].

Earlier reports of molecular markers of liver cancer in a mouse model were examined by *ex vivo* manipulation of progenitor cells followed by their transplantation into recipient mice [4–6]. While these studies initiated with progenitor cells harboring cancer-predisposing lesions identified valuable markers of liver cancer, they do not model HCV infection-associated HCC.

Unlike the expression of uPA transgene under albumin promoter (Alb-uPA) as originally described [7], MUP-uPA/SCID/Bg mice studied in this report, provide a long window of time (up to 12 months) for hepatocyte engraftment and efficient infection with HBV or HCV [3]. We observed primary liver cancers within the engrafted human liver in about 25 % HCV infected mice, at four to six months post-infection.

To identify molecular markers of HCV infection-associated HCC, we compared expression levels of oncoproteins and tumor suppressor proteins from liver tissues of HCV-infected HCC with the HCV-infected but HCC-negative mice. Results suggest that loss of PTEN tumor suppressor protein is an early indicator of HCV infection-associated HCC. By contrast, induction of c-Myc and inflammatory response molecules, correlate with HCC progression.

Micro-RNAs have been studied as independent markers of oncogenic progression [8]. In comparison with miRNAs reported from liver cancer of unspecified origin, our analysis suggests that induction of oncomiRs (miR-21, miR-221 and miR-141) and tumor suppressor miR-26a constitute signature miRNA markers of HCV infection-associated HCC. Overall, the results indicate that human hepatocyte engrafted MUP-uPA/SCID/Bg mice are a suitable small animal model for studying HCV-infected HCC and the role of tumor-promoting factors in liver cancer.

## Methods

### Scheme for generation of humanized mice

Procedures for human hepatocyte engraftment and HCV infection of immune compromised (MUP-uPA/SCID/Bg) mice were described earlier [3]. Eleven of 42 HCV-infected mice developed tumors four to six months post-infection; the controls, 21 mice that were engrafted but not infected, or 23 mice that were not engrafted, did not develop tumors after being followed for the same period. Histologic changes similar to human hepatocellular carcinoma were observed within enografted human liver of HCV-infected mice; as well, the tumors stained for human albumin and human glypican-3 (Tesfaye et al, unpublished).

### Protein extraction, subcellular fractionation and immunoblotting

Liver tissues used for Western blot analysis were from human hepatocyte-engrafted MUP-uPA/SCID/Bg uninfected control, or HCV-infected but HCC-negative or HCV infection-induced liver tumors (HCC positive) mice.

Liver samples were stored at -80 °C until analysis. Liver tissues were lysed with RIPA buffer supplemented with protease inhibitor cocktail (Roche) as described [9]. Cell fractionation kit (Thermo Pierce) was used for the fractionation of nuclear and cytoplasmic proteins. Briefly, 200 μL Cytoplasmic Extraction Reagent (CER I) was used to homogenize liver tissues followed by the addition of 11 μL Cytoplasmic Extraction Reagent II (CER II) to precipitate the nuclear fraction. 100 μL of Nuclear Extraction Reagent (NER) was used to extract the nuclear fraction. Equal amounts (10 μg) of protein fractions were analyzed on 10 % precast Mini-PROTEIN gel (BIO-RAD). Proteins were transferred to PVDF membrane, blocked with 5 % non-fat milk and probed with antibodies (Abcam) as indicated. HRP conjugated anti-rabbit or anti-goat antibodies (Abcam; SuperSignal West Dura Chemiluminescent Substrate) were visualized with BIO-RAD ChemiDoc™ XRS+ System. Estimates of relative protein levels were based on β-actin as internal (loading) control. Western blot images were quantified (by Image Lab™ Software developed by BIO-RA) as described earlier [9–11]. Statistical analysis was based on paired *t*-test for each subgroup compared with the reference (loading controls). Data shown is SE of the mean (SEM). Statistical significance is set to *p < 0.05.

### Real-time quantitative PCR of RNA samples from chimeric mouse liver

1ug of total RNA was added to QuantiMir RT Kit Small RNA Quantification System (System Biosciences) following the manufacturer’s protocol. The reverse-transcribed product was then diluted 40 fold. Real time quantitative PCR was performed with iTaq SYBR Green Supermix with Rox (Bio-Rad) on ABI 7300 PCR system. Primers used for detecting hsa-miR-141, hsa-miR-23a, hsa-miR-122, hsa-miR-181, hsa-miR-21, hsa-miR-221, hsa-miR-26a and hsa-miR-16 respectively were as follows: 5’-TAACA CTGTC TGGTA AAGAT GG-3’, 5’-ATCAC ATTGC CAGGG ATTTC C-3’, 5’-TGGAG TGTGA CAATG GTGTT TG-3’, 5’-AACAT TCAAC GCTGT CGGTG AGT-3’, 5’-TAGCT TATCA GACTG ATGTT GA-3’, 5’-AGCTA CATTG TCTGC TGGGT TTC-3’, 5’-TTCAA GTAAT CCAGG ATAGG CT-3’ and 5’-TAGCA GCACG TAAAT ATTGG CG-3’

Statistical analysis: All quantitative real-time PCR and densitometry data were analyzed using Microsoft Excel 2010, using miR-16 (which does not change following HCV infection) for normalization.

## Results

### Inactivation of tumor suppressor and amplification of oncogene

Loss of tumor suppressor proteins and induction of oncogenic proteins are the initiating events that promote deregulation of molecular pathways underlying the development of hepatocellular carcinoma [12, 13]. We investigated whether HCV infection associated HCC is characterized by the loss of tumor suppressor proteins. Of particular interest is the deregulation of PTEN tumor suppressor due to its critical role in hepatocellular carcinoma [14–16]. Viral role in PTEN depletion was suggested in an earlier report which showed that HCV-derived small non-coding RNA restricted nuclear translocation of PTEN protein by down-regulating Transportin 2 expression in HCV-infected human hepatocytes [9]. Here we investigated whether the loss of PTEN defines HCC progression in a humanized mouse model of HCV infection.

**Fig. 1:**
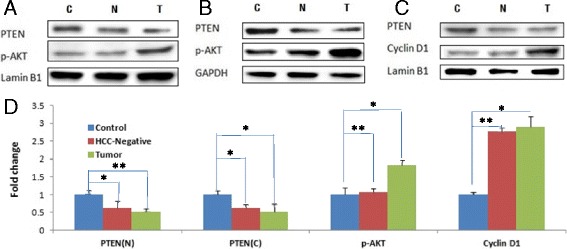
Tumor suppressor proteins: Liver tissues from control animals (MUP-uPA/SCID/bg mice engrafted with human hepatocytes but not infected with HCV), HCC-negative mice (engrafted and HCV infected mice that did not develop HCC), and HCC positive mice (engrafted and HCV infected) were examined by Western blotting with monoclonal antibodies against PTEN, Phospho-Akt or Cyclin D1. Panels (**a**) and (**c**) show representative Western blots of nuclear protein fraction (with Lamin B1 as loading control); and panel (**b**) is representative Western blot of corresponding cytoplasmic fraction (with GAPDH as loading control). Liver tissues were homogenized with RIPA buffer, and 30 microgram total proteins per lane were resolved by SDS-PAGE, and immunoblotted with antibodies as described before [11]. Panel (**d**) represents quantitative assessment (fold change) of nuclear or cytoplasmic PTEN (PTEN-N, PTEN-C); cytoplasmic p-Akt or nuclear Cyclin-D1 from the liver tissues. Quantitation was based on 12 controls, 7 HCC negative and 13 HCC positive liver tissues, analyzed in three independent SDS-PAGE runs. The Western blots were normalized to the internal “loading” controls (Lamin-B1 for nuclear and GADH for cytoplasmic fractions) (*p < 0.05)

Immunoblots of PTEN protein from liver tumors (T) of thirteen hepatocyte-engrafted and HCV-infected MUP-uPA/SCID/bg mice and twelve control (C) mice that were engrafted with human hepatocytes but not infected with HCV are shown Fig. 1. We also examined liver tissues from seven MUP-uPA/SCID/bg mice that were engrafted with human hepatocytes and infected with HCV but remained HCC-negative (N). We observed a consistent decline of both nuclear (Fig. 1a and c) and cytoplasmic (Fig. 1b) PTEN protein in HCV-infected HCC. Interestingly, PTEN protein in HCV-infected but HCC-negative liver (N) also declined to similar extent (Fig. 1d), suggesting that loss of PTEN may be necessary but insufficient to promote HCC.

The next issue is whether the PTEN produced in chimeric mouse liver is functional. PTEN is a dual specificity phosphatase with lipid and protein phosphatase activities [17–19]. Cytoplasmic lipid phosphatase activity of PTEN is a central negative regulator of phosphatidylinositol-3-kinase (PI3K) signal cascade for cell growth and proliferation. Loss of PTEN is associated with increased phosphorylation of Akt, a proto-oncogene with key roles in cell survival and cell proliferation in many types of cancer [15, 16]. We observed increased phosphor-Akt levels in HCV-infected liver tumor (Fig. 1a, b and d), consistent with the role of PTEN as a tumor suppressor.

Nucleus-localized PTEN protein has an essential role in cell cycle homeostasis and genomic stability [17, 19, 20]. We observed a reduced level of nuclear PTEN that corresponded with increased Cyclin D1 in HCV infection-associated liver tumors (Fig. 1c). The observed increase in Cyclin D1 in liver tumors is consistent with enhanced proliferation of HCV-infected hepatocytes [10]. Interestingly, we observed similar increase in Cyclin D1 of HCV-infected but HCC-negative liver (Fig. 1c and d), suggesting that deregulation of the cell cycle is an early event resulting from PTEN insufficiency of HCV-infected cells.

### Induction of c-Myc oncoprotein

**Fig. 2:**
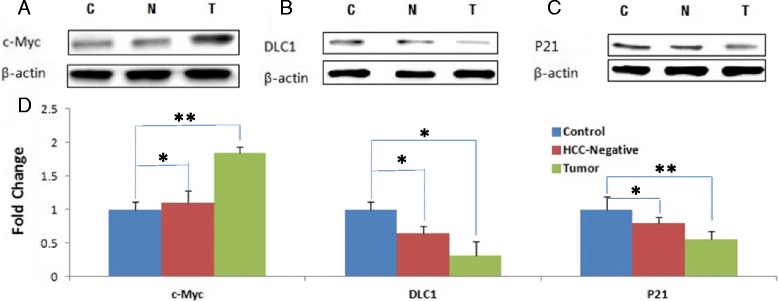
Oncoproteins: Western blots of the control, HCC-negative and liver tumor tissues (as is shown in Fig. 1) were probed with antibodies against c-Myc, DLC-1 or p21 proteins (panels **a**, **b** and **b**). Panel (**d**) represents quantitative analysis (based on the loading controls) of liver tissues from uninfected control, HCC negative and HCC positive mice (as in Fig. 1)

c-Myc is a constitutively induced transcription activator in a broad range of human cancers [21, 22]. We observed increased c-Myc protein levels in HCV-infected liver tumors compared to the control (Fig. 2a). By contrast, induction of c-Myc in HCC-negative liver was modest (Fig. 2d), suggesting that induction of c-Myc oncoprotein is a relatively late event in the development of HCV-infection associated HCC.

### Down-regulation of DLC-1 tumor suppressor

DLC1 (Deleted in Liver Cancer 1) gene maps to chromosome 8 (8p21.3-22), a region that is frequently deleted in solid tumors. DLC1 encodes GTPase-activating protein (GAP) that acts as a negative regulator of the Rho family GTPases [23]. Loss of DLC1 is considered an independent marker of hepatocellular carcinoma [4, 6]. We observed the loss of DLC1 protein in HCV-infected liver tumors and less so in HCV-infected but HCC-negative liver tissue (Fig. 2b and d).

### P21

HCV structural and non-structural proteins have been shown to interact with and modulate transcriptional regulatory activity of p53 tumor suppressor protein [24–26]. Modulation of transcriptional activity of p53 by viral proteins distinguishes HCV from other RNA viruses in its ability to interfere with the p53 function [13]. Therefore, it was important to determine if HCV infection of humanized mice modulated p53 to promote HCC. We assessed the modulation of p53 function in HCV-infected chimeric mice on the basis of p21 expression, a direct target of p53 transcriptional regulatory function. We observed a marked decline of p21 protein in HCV-infected liver tumor (Fig. 2c and d), and less so in HCC-negative mice (N, Fig. 2d), suggesting that HCC progression is correlated with the loss of function of p53 tumor suppressor.

### Inflammatory response

**Fig. 3:**
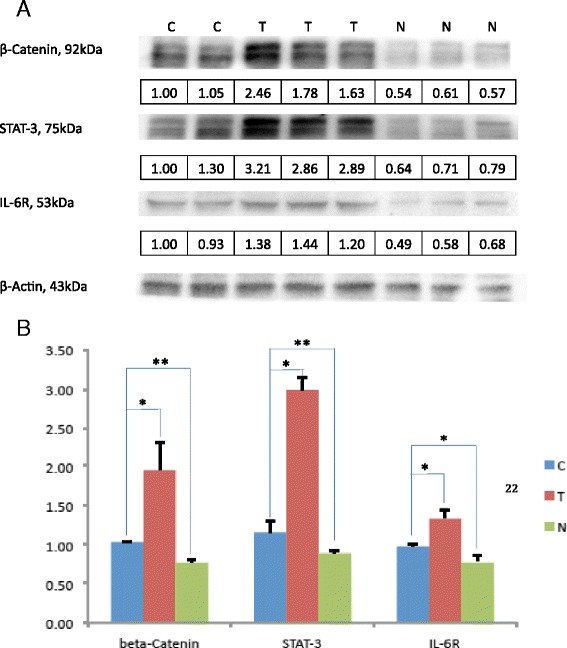
*Inflammatory response markers:* Total proteins from liver tissues of 7 uninfected controls, 8 HCV infected HCC negative and 7 HCV infected HCC positive mice were analyzed by Western blotting. (Fig. 3a). Representative Western blots of two controls (C), three HCC (T) and three HCC negative (N) liver are shown. Numbers underneath is relative values normalized to β-Actin loading control. (Fig. 3b): Quantitative assessment of β-Catenin, STAT-3 and IL-6R (from 7 uninfected control, 8 HCC negative and 7 HCC positive mice) was based on B-Actin internal controls analyzed by three independent SDS-PAGE runs

Persistent viral infection is an underlying cause of inflammation-induced cancer, including HCC. More than 90 % of HCCs arise in the context of hepatic injury and inflammation. Inflammation-associated oncogenic response is mediated by STAT proteins; in particular, activated STAT3 [27, 28]. To ascertain if HCV infection-associated HCC in humanized mice mimics the natural inflammatory response, we assayed activated STAT3 levels in the liver tumors and in HCC-negative as compared to the uninfected control mice. As shown (Fig. 3), there is a marked induction of activated (phosphorylated) STAT3 in HCV infection-associated liver tumors as compared to HCV-infected but HCC-negative liver.

Next, we investigated whether the activation of STAT3 in liver tumors was coordinately regulated with other inflammatory response proteins, notably interleukin-6 (IL-6) and IL-6R [28–30], in HCV-infected chimeric mice. As shown in Fig. 3, we observed a parallel increase in STAT3 and IL-6R in HCV-infected HCC as compared with control liver, suggesting coordinate regulation of inflammatory response molecules in tumor development.

Signal transduction pathways, in particular *Wnt* signaling, are frequently mutated in liver cancer. Disruption of *Wnt* signaling results in β-Catenin stabilization and translocation to the nucleus where it activates cell survival and cell proliferation genes [12]. Activation of β-Catenin in HCC as contrasted with the surrounding (normal) liver tissue was recently reported from HCV-infected patients [31]. Considering the significance of β-Catenin activation and Wnt signaling in human cancer, we considered it important to investigate the activated state of these tumorigenic proteins in HCV infection-associated HCC in humanized mice. Results illustrated in Fig. 3a, b show coordinate regulation of inflammatory response proteins and deregulation of signal transduction pathways (activated β-Catenin, IL-6R and STAT3) in HCV-infected HCC.

### MicroRNA markers of HCC

MicroRNAs can function as tumor suppressors or oncogenes (oncomiRs) [32]. Altered expression levels of miRNAs have been reported in a number of human cancers [8, 32–34]. In this study we sought to identify miRNAs that would serve as distinguishing markers of HCV infection-associated HCC.

**Fig. 4:**
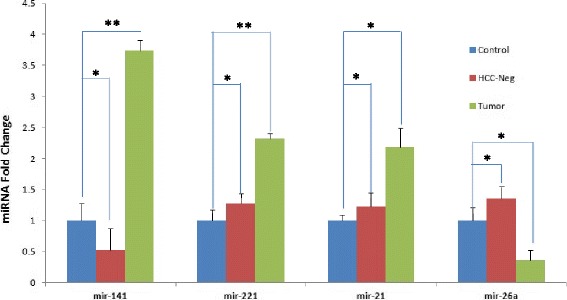
*Altered MicroRNA expression*: Changes in microRNA expression in Controls, HCC negative and Liver Tumor tissues were analyzed by RT-PCR. Total RNA was prepared by Trizol procedure and equal RNA amounts were analyzed by qRT-PCR. The data represents similar number of Controls, HCC negative and HCC positive liver tissues (as in Fig. 1), analyzed in triplicates

MicroRNA 141 (miR-141) is induced in HCV-infected human primary hepatocytes. Importantly, miR-141 directly targets DLC1 tumor suppressor protein expression [10], attesting to its role as bona fide oncomiR. Here we compared expression levels of miR-141 along with other known oncomiRs (miR-21 and miR-221) in HCV infection-induced HCC (Fig. 4). Results suggest that expression of miR-141 and oncomiRs miR-21 and miR-221 that target cell cycle inhibitors [34, 35] is coordinately induced in HCV infection-associated HCC.

Tumor suppressor microRNA 26a (miR-26a) is depleted in liver tumor as compared to the surrounding normal liver tissues [36]. We then investigated whether the HCV-infected HCC in our humanized mouse model down regulated miR-26a tumor suppressor, resembling the loss of miR-26a in human HCC. We observed a marked reduction of miR-26a in liver tumors of human hepatocyte-engrafted HCV-infected mice compared to controls (Fig. 4). Our results suggest that induction of oncomiRs (miR-141, miR-21 and miR-221) and down-regulation of tumor suppressor miRNA (miR-26a) constitute distinguishing markers of HCV infection-associated HCC.

**Fig. 5:**
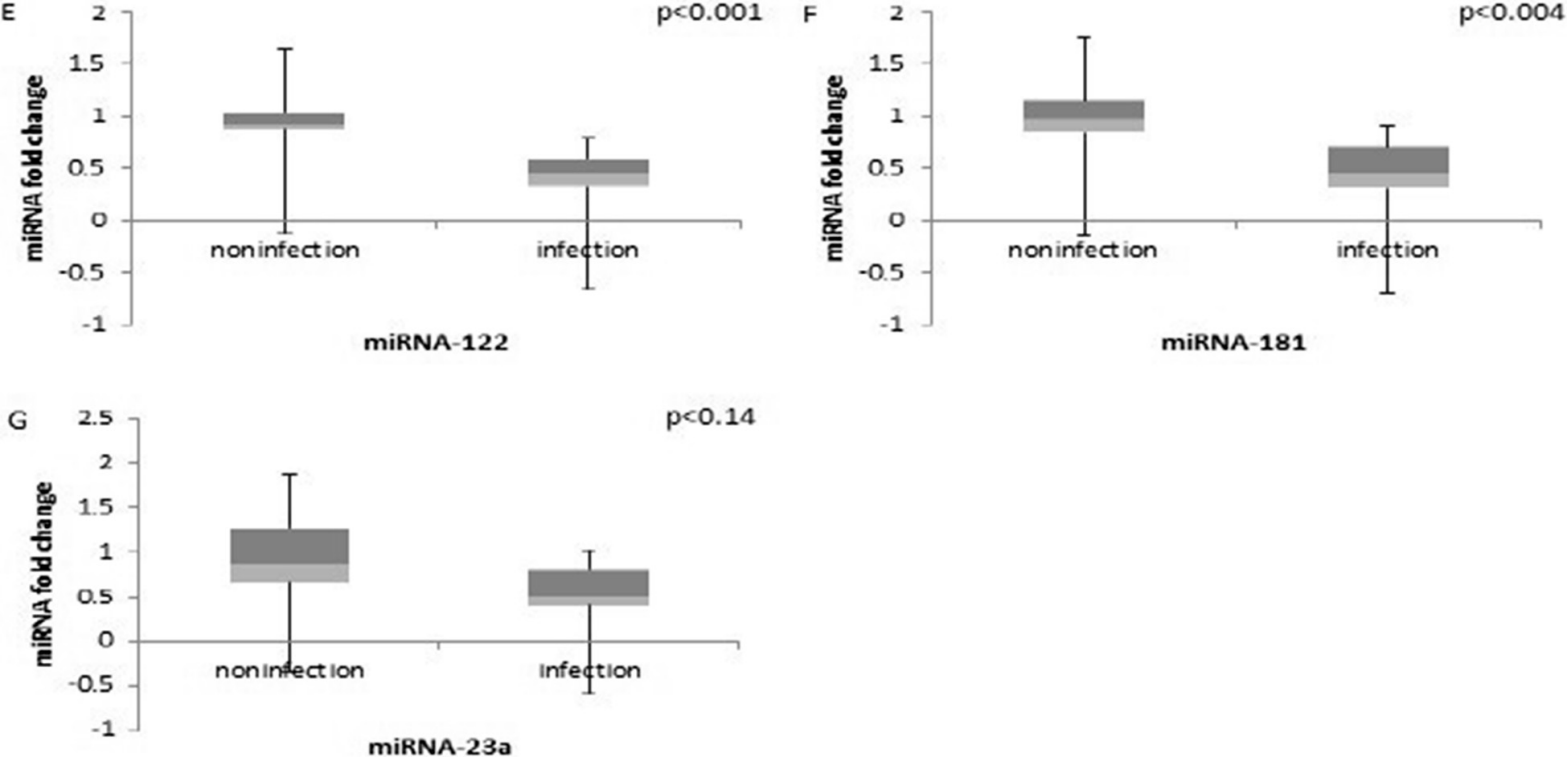
*HCV non-specific microRNAs:* Total RNA *f*rom eight HCV infected and *four* uninfected human hepatocyte engrafted MUP-uPA/SCID/Bg mice were analyzed by qRT-PCR. Relative values of microRNAs are shown as fold change compared to the uninfected control liver

Altered expression levels of miRNAs have also been reported from liver tumors of heterogeneous origin. To identify changes in miRNA expression that distinguish HCV infection-associated HCC, we examined selected examples of miRNAs that have been reported from studies of various liver tumors. Increase in miR-181 levels was reported in AFP-positive tumors and in embryonic liver cells [37]. Our results by contrast, showed a decline of miR-181 in HCV infected-HCC (Fig. 5), suggesting that miR-181 is less reliable indicator of HCV infection-associated liver tumors.

Contribution of miR-122 in HCV replication has been studied in a number of cell culture systems and in animal models; however, its role in HCV infection-related HCC in humans is less clear [38, 39]. A recent report [40] suggests that phenotypic effects of miR-122 depletion may be a consequence of its sequestration by HCV genomic RNA. Our analysis of miR-122 shows an insignificant change in HCV infection-associated HCC of human hepatocyte-engrafted mice (Fig. 5).

MicroRNA 23a (miR-23a) has been shown to target genes involved in gluconeogenesis at different stages of hepatocarcinogenesis in mouse liver as well as in primary human HCC [41, 42]. In our studies, amplification of miR-23a appears to be a less specific indicator of HCV infection-induced HCC of humanized mice. As shown (in Fig. 5), liver tumors of HCV-infected MUP-uPA/SCID/bg mice show a relatively modest change in miR-23a levels.

Our studies suggest that amplification of oncomiRs (miR-21, miR-221 and miR-141) and the loss of tumor suppressor miR-26a are specific indicators of HCV infection-associated HCC. By contrast, altered expression of miR-181, miR-122 and miR-23a appears to be a less specific indicator of HCV infection-associated HCC.

## Discussion

In this study of a mouse model of HCV infection-associated liver tumor, we have attempted to identify tumor suppressor genes and miRNAs whose expression defines HCV infection-associated HCC. PTEN is a haploinsufficient tumor suppressor gene [43]; single functional allele of PTEN is insufficient to maintain normal function [44–48]. In particular, insufficiency of nuclear PTEN has been shown to result in genomic instability [49, 50]. HCV utilizes a novel mechanism to induce PTEN insufficiency, involving viral non-coding RNA directed post-trasncriptional silencing of Transportin-2 that restricts translocation of PTEN protein to the nucleus [9]. The results of PTEN depletion in liver tumors of HCV-infected humanized mice described here are consistent with *in vitro* studies of HCV-infected human hepatocytes reported earlier [9]. Comparison of PTEN protein levels of HCV-infected but HCC-negative liver with the HCV-infected HCC suggest that loss of PTEN is an early, precancerous event, although PTEN insufficiency by itself does not promote HCC. Similarly, loss of DLC-1 tumor suppressor protein appears to be an early indicator of HCV infection-associated HCC. By contrast, induction of oncogenic modulators such as cMyc, miR-21, miR221 and miR-141 appear to be effective in promoting HCC progression.

Induction of inflammatory effector molecules in HCV infection-associated HCC described here support the notion that an inflammatory microenvironment is an essential factor in the development of HCC. Further studies with the humanized mouse model are needed to address the issue of whether the oncogenic changes induced early in HCV infection promote the sustained inflammatory microenvironment required for tumor progression [28]. A summary of altered expression levels of tumorigenic markers during HCC progression is described in Table 1.

**Table 1:** Schematic representation of the relative changes in oncoproteins or Tumor suppressor proteins, and microRNAs in HCV-infected HCC negative (N) and liver tumors (T) as compared with the control liver (derived from the results shown in Figs. 1, 2, 3, 4 and 5).

		Levels	
Category	Control	HCC(Neg)	HCC
Tumor Suppressor			
PTEN(N)	+++	++	++/+
PTEN (C )	+++	++	++/+
DLC1	+++	++	+
p21	++	++	+
Oncoprotein			
p-AKT	++	++	+++
CyclinD1	+	+++	+++
c-myc	+	+	+++
STAT3	+	+	+++
OncomiR			
miR-141	++	+	++++
miR-221	+	+	+++
miR-21	+	+	+++
Tumor Suppressor(miRNA)			
miR-26a	++	+++	+

Induction cMyc oncoprotein is more pronounced in HCV-infected liver tumors as compared to HCC-negative liver (Fig. 2a and d), suggesting that the induction of cMyc oncoprotein is a relatively late event in the development of hepatocellular carcinoma. By contrast, down-regulation of tumor suppressor proteins PTEN, DLC-1 and p21 appears to be initiated early in HCV infection-associated HCC.

### Induction of oncomiRs and inhibition of tumor suppressor microRNA

MicroRNAs (miRNAs) represent a substantial fraction of tissue-specific small non-protein coding RNA modulators of gene expression. MicroRNAs can function as gene silencers by blocking mRNA translation and destabilizing the target mRNA. Phenotypic consequences of miRNA-regulated genes evoke essential features of tumor biology, including modulation of apoptosis, cell proliferation, signal transduction and stress response. Dysregulated expression of miRNAs has proven valuable in tumor classification and prognosis [8, 34, 35]. In this study we have presented evidence of induction of tumor-promoting microRNAs, oncomiRs miR-141, miR-21 and miR-221, and down-regulation of tumor suppressor microRNA, miR-26a, that represent distinguishing features of HCV infection-associated HCC. Future studies are needed to understand how coordinate regulation of miRNA target genes promote the development of HCV infection-associated HCC.

Tumor suppressor genes modulate signaling networks that protect against tumor initiation and progression. Loss of function of tumor suppressor genes (TSGs) is generally due to inactivation by deletions, point mutations or promoter hypermethylation. We present evidence of down-regulation of tumor suppressor proteins in a humanized mouse model of HCV infection-associated HCC. These studies represent the first example of functional insufficiency of tumor suppressor proteins induced by HCV infection. The studies with a humanized mouse model of HCC should encourage investigation of molecular pathways that are modulated by amplification of oncogenes and the inhibition of tumor suppressor genes.

## Abbreviations

Akt: Protein Kinase B, a serine threonine-specific protein kinase that plays a key role in cell proliferation, transcription and migration
Alb-uPA: Albumin promoter driving urokinase plasminogen activator transgene
cMyc: Proto-onco gene that codes for transcription factor named derived from Avian Myelocytoma viral oncogene)
Cyclin D1: Cell cycle kinase co-activator
DLC-1: Deleted in Liver Cancer1 (DLC1) gene encodes a GTPase activating protein that functions as a negative regulator of Rho family of GTPases
Haploinsufficiency: wherein the retained functional copy of a gene is insufficient to maintain normal function
HBV: Hepatitis B virus
HCC: Hepatocellular carcinoma
HCV: Hepatitis C virus
IL-6R: Interleukin 6 receptor
MUP-uPA/SCID/Bg: Major Urinary Protein promoter driving urokinase plasminogen activator transgene in severe combined immune deficient mice, Bg strain
oncomiR: Oncogenic microRNA
p53: (53kda) tumor suppressor gene
PI3K: Phosphatidylinositol-3-kinase
PTEN: Phosphatase and tensin homologue deleted on chromosome 10
RT-PCR: Reverse transcriptase-polymerase Chain Reaction
STAT3: Signal transducer and activator of transcription protein 3 with inflammation-related oncogenic potential
Wnt: Oncogenic signaling proteins, described in mouse mammary tumor virus originally characterized from *Drosophila Wingless (g)* gene
β-Catenin: Encoded by CTNB1 gene in humans, a dual function protein regulating cell-cell adhesion and transcription overexpression of β-Catenin associated with many cancers including hepatocellular carcinoma
